# Interleukin-22 receptor 1-mediated stimulation of T-type Ca^2+^ channels enhances sensory neuronal excitability through the tyrosine-protein kinase Lyn-dependent PKA pathway

**DOI:** 10.1186/s12964-024-01688-6

**Published:** 2024-06-03

**Authors:** Hua Cai, Siyu Chen, Yufang Sun, Tingting Zheng, Yulu Liu, Jin Tao, Yuan Zhang

**Affiliations:** 1 Clinical Research Center of Neurological Disease, Department of Geriatrics, The Second Affiliated Hospital of Soochow University, Suzhou, 215004 P.R. China; 2 Department of Physiology and Neurobiology & Centre for Ion Channelopathy, Suzhou Medical College of Soochow University, Suzhou, 215123 P.R. China; 3 Jiangsu Key Laboratory of Neuropsychiatric Diseases, Soochow University, Suzhou, 215123 P.R. China; 4 MOE Key Laboratory of Geriatric Diseases and Immunology, Suzhou Medical College of Soochow University, Suzhou, 215123 P.R. China

**Keywords:** Interleukin 24, Trigeminal ganglion neurons, Pain, T-type Ca^2+^ channels, Tyrosine-protein kinase Lyn

## Abstract

**Background:**

Interleukin 24 (IL-24) has been implicated in the nociceptive signaling. However, direct evidence and the precise molecular mechanism underlying IL-24’s role in peripheral nociception remain unclear.

**Methods:**

Using patch clamp recording, molecular biological analysis, immunofluorescence labeling, siRNA-mediated knockdown approach and behavior tests, we elucidated the effects of IL-24 on sensory neuronal excitability and peripheral pain sensitivity mediated by T-type Ca^2+^ channels (T-type channels).

**Results:**

IL-24 enhances T-type channel currents (T-currents) in trigeminal ganglion (TG) neurons in a reversible and dose-dependent manner, primarily by activating the interleukin-22 receptor 1 (IL-22R1). Furthermore, we found that the IL-24-induced T-type channel response is mediated through tyrosine-protein kinase Lyn, but not its common downstream target JAK1. IL-24 application significantly activated protein kinase A; this effect was independent of cAMP and prevented by Lyn antagonism. Inhibition of PKA prevented the IL-24-induced T-current response, whereas inhibition of protein kinase C or MAPK kinases had no effect. Functionally, IL-24 increased TG neuronal excitability and enhanced pain sensitivity to mechanical stimuli in mice, both of which were suppressed by blocking T-type channels. In a trigeminal neuropathic pain model induced by chronic constriction injury of the infraorbital nerve, inhibiting IL-22R1 signaling alleviated mechanical allodynia, which was reversed by blocking T-type channels or knocking down Cav3.2.

**Conclusion:**

Our findings reveal that IL-24 enhances T-currents by stimulating IL-22R1 coupled to Lyn-dependent PKA signaling, leading to TG neuronal hyperexcitability and pain hypersensitivity. Understanding the mechanism of IL-24/IL-22R1 signaling in sensory neurons may pave the way for innovative therapeutic strategies in pain management.

**Supplementary Information:**

The online version contains supplementary material available at 10.1186/s12964-024-01688-6.

## Introduction

Interleukin-24 (IL-24), a conserved member of the IL-10 family of cytokines, is broadly expressed in a variety of immune cells including B cells, macrophages and monocytes, as well as in brain and peripheral nervous tissues of mammals [1]. IL-24 shares a 20–30% amino acid homology with other IL-10 family cytokines including IL-10, IL-20, and IL-22 and interacts with two different heterodimeric receptor complexes, IL-20R1/IL-20R2 and IL-22R1/IL-20R2 [2]. Acting through its membrane receptors, IL-24 serves various crucial biological functions in the pathogenesis of neurological diseases including but not limited to psoriasis, inflammatory bowel disease, rheumatoid arthritis and multiple sclerosis [3–5]. Furthermore, recent evidence has highlighted the potential role of IL-24 in nociceptive pain behaviors. Specifically, studies have shown an upregulation of IL-24 levels in either the spinal dorsal horn or dorsal root ganglia in a rat model of spared nerve injury [6]. Additionally, it has been demonstrated that intrathecal injection of IL-24 or the IL-20R2 neutralizing antibody effectively relieved mechanical allodynia [6]. Moreover, IL-24 treatment was also shown to increase β-endorphin levels in plasma of animals to relieve cancer pain [7]. Nevertheless, the involvement of IL-24 in peripheral nociceptive responses and the specific mechanisms by which it participates remain unknown.

T-type Ca^2+^ channels (T-type channels), characterized by their low-voltage activation and distinctive ability to modulate neuronal excitability with minimal depolarization, have been shown to play a pivotal role in controlling low-threshold exocytosis [8, 9]. The cloning of the α1 subunits of T-type channels has revealed three distinct subtypes, known as Cav3.1, Cav3.2, and Cav3.3, each with specific expression patterns and unique pharmacological profiles in both the brain and peripheral organs [10, 11]. Disregulated expression and/or function of these Cav3 channels have been linked to pathological conditions such as seizure susceptibility, slow-wave sleep, absence epilepsy, and pain perception [12, 13]. In the peripheral pain processing, modulation of T-type channels may not only influence the firing patterns and action potential frequency of sensory neurons, but play a crucial role in neurotransmitter releasing at dorsal horn synapses [14–16]. Growing evidence from pharmacological analyses [17] and genetic [18] suggests significant therapeutic potential in targeting T-type channels for pain management.

In this study, we investigated the impact of IL-24 on the regulation of TG T-type channels and elucidated the molecular mechanisms through which IL-24 acts as a nociceptive effector. Our results indicate that IL-24 activates T-type channels via IL-22R1, which is linked to a novel Lyn-dependent PKA signaling, independent of cyclic adenosine monophosphate (cAMP). The IL-22R1-mediated signaling cascade contributes to membrane hyperexcitability in TG neurons and the manifestation of nociceptive behaviors in mice. Targeting IL-22R1-mediated signaling may present novel therapeutic strategies and targets for pain management.

## Materials and methods

### Dissociation of TG neurons

All experimental procedures conducted in this study received approval from the Institutional Animal Care and Use Committee of Soochow University and were carried out in strict adherence to the National Institutes for Health guidelines for the use of laboratory animals. Trigeminal ganglion (TG) neurons extracted from ICR mice, which were 6 to 8 weeks old regardless of sex, were dissociated following the methods described in previous publications [19–21]. In brief, TGs were finely chopped into small pieces and digested for 30 min with 1.5 mg/ml collagenase (Roche) and 20-minute incubation with 1.25 mg/ml trypsin (Sigma-aldrich) at 37 °C. Subsequently, TG neurons were subjected to mechanical trituration using three fire-polished Pasteur pipettes with decreasing diameters. The dissociated cells were then plated on Matrigel-coated 12-mm glass cover slips. To efficiently group the TG neurons, we classified them into two distinct categories based on their soma diameter: small-sized neurons (soma diameter less than 25 μm) and medium-sized neurons (soma diameter between 25 and 35 μm). Electrophysiological recordings of small-sized neurons were performed within a time window of 3 to 6 h after plating.

### Electrophysiology

Following the protocols as previously described, patch clamp experiments were conducted under room temperature conditions using a standard whole-cell recording setup [19, 22]. Currents were low-pass filtered at 1 kHz (Digidata 1440) and sampled at 50 kHz [23]. Pipettes were fabricated from borosilicate glass (1.5 mm outer diameter, 0.86 mm inner diameter; Sutter Instruments), and had a resistance between 3 and 5 MΩ when filled with internal solution. Membrane currents were recorded utlizing pClamp 10.2 software from Molecular Devices and the series resistance was compensated at least 75%. For measuring the T-currents, the patch pipettes were filled with an intracellular solution that consisted of the following (in mM): 110 CsCl, 0.3 Na_2_GTP, 4 Mg-ATP, 25 HEPES, and 10 EGTA, and was adjusted to pH 7.4 and an osmolarity of 295 mOsm. The external solution consisted of the following (in mM): 140 TEA-Cl, 5 CsCl, 0.5 MgCl_2_, 5 BaCl_2_, 5.5 D-glucose and 10 HEPES, and was adjusted to pH 7.4 and an osmolarity of 305 mOsm. This solution was adjusted to pH 7.3 and an osmolarity of 295 mOsm. An extracellular bath solution, composed of 5 µM nifedipine (a blocker for L-type channels), 0.2 µM ω-conotoxin MVIIC (a blocker for N-type and P/Q-type channels), and 0.2 µM SNX-482 (a blocker for R-type channels), was utilized during the recordings of isolated T-currents. For measuring the voltage-gated K^+^ channel (Kv) current, the patch pipettes were filled with an intracellular solution that consisted of the following (in mM): 140 KCl, 10 HEPES, 5 EGTA, 3 Mg-ATP, 1 MgCl_2_, 0.5 CaCl_2_, and 0.5 Na_2_GTP, and was adjusted to pH 7.4 and an osmolarity of 295 mOsm. The external solution used for Kv current recording consisted of the following (in mM): 5 KCl, 1 MgCl_2_, 0.03 CaCl_2,_ 150 choline-Cl, 10 HEPES, and 10 D-glucose, and was adjusted to pH 7.4 and an osmolarity of 310 mOsm. During current-clamp or Nav current recordings, patch pipettes were filled with an intracellular solution that consisted of the following (in mM): 110 KCl, 25 HEPES, 10 NaCl, 0.3 Na2GTP, 4 Mg-ATP, and 2 EGTA, and was adjusted to pH 7.4 and an osmolarity of 295 mOsm. The extracellular solution was consisted of 125 mM NaCl, 2 mM KCl, 2 mM CaCl_2_, 2 mM MgCl_2_, 30 mM D-glucose and 25 mM HEPES, and was adjusted to pH 7.4 and an osmolarity of 305 mOsm. For the application of IL-24 to a patched neuron, an air-pressure system called the Pneumatic PicoPump (PV830, World Precision Instruments) was utilized. In order to deliver compounds intracellularly, the electrode pipettes had resistances ranging from 2 to 3 MΩ. IL-24 was administered to a patched neuron using an air-pressure microinjector (PV830 Pneumatic Picopump, World Precision Instruments) with a glass pipette, positioned 15–25 μm away from the soma of TG neurons. In siRNA knockdown experiments, patch clamp recordings were performed specifically on small-sized TG neurons that exhibited green fluorescence.

### Western blot analysis

Immunoblot analysis was conducted as previously described [19, 24–26]. Briefly, trigeminal ganglia were removed, dissected, and homogenized in radioimmunoprecipitation assay buffer (RIPA) buffer in the presence of proteinase inhibitor cocktail. Lysates were then centrifuged at 4 °C to remove insoluble material. Protein concentration was then determined using the BCA method (BCA Protein Assay Kit, Thermo Scientific). Samples were loaded into 10% sodium dodecyl sulfate-polyacrylamide gel electrophoresis (SDS-PAGE) gels and transferred onto polyvinylidene difluoride (PVDF) membranes. The membrane was blocked with 5% skim milk and incubated overnight at 4 °C with the following antibodies: IL-20R1 (mouse, 1:1000, Santa Cruz, Cat. No. sc-80,065), IL-22R1 (rabbit, 1:1000, ProteinTech, Cat. No. 13462-1-AP), JAK1 (mouse, 1:1000, ProteinTech, Cat. No.66466-1-IG), *p*-JAK1 (rabbit, 1:1000, Abcam, Cat. No. ab138005), ERK (rabbit, 1:1000, Cell Signaling Technology, Cat.No. #4695), *p*-ERK (rabbit, 1:1000, Cell Signaling Technology, Cat. No. #4370), p38 (rabbit, 1:1000, Cell Signaling Technology, Cat. No. #4695), *p*-p38 (rabbit, 1:1000, Cell Signaling Technology, Cat. No. #4511), JNK (rabbit, 1:1000, Cell Signaling Technology, Cat. No. #9252), *p*-JNK (rabbit, 1:1000; Cell Signaling Technology, Cat. No. #4668), Lyn (mouse, 1:1000, ProteinTech, Cat. No. 60211-1-IG), *p*-lyn (rabbit, 1:1000, Abcam, Cat. No. ab33914), PKA (rabbit, 1:1000, Cell Signaling Technology, Cat. No. #4782), *p*-PKA (rabbit, 1:1000, Abcam, Cat. No. ab75991), Cav3.2 (mouse, 1:1000, Novus, Cat. No. NBP1-22444) and GAPDH (rabbit, 1:10000, ProteinTech, Cat. No. 10494-1-AP). Blots were washed and subsequently probed with appropriate horseradish peroxidase-conjugated secondary antibodies [27]. Enhanced chemiluminescence (Merck Millipore) was utilized to detect the immunocomplexes. The bands were detected using the Chin-X Imager System (Shanghai, China), and the protein band intensities were quantified using NIH ImageJ software.

### Immunohistochemistry

Immunostaining analysis was performed following previously described methods [19, 25]. The trigeminal ganglia (TGs) were sliced into 15-µm sections using a cryostat (Leica CM1950) and treated with 0.2% Triton X-100 to permeabilize them. Subsequently, the sections were blocked with 5% goat serum, washed with PBS, and incubated with primary antibodies against IL-22R1 (rabbit, 1:200, ProteinTech, Cat. No. 13462-1-AP), NeuN (mouse, 1:200, Cell Signaling Technology, Cat. No. #94,403), glutamine synthetase (GS, mouse, 1:200, Abcam, Cat. No. ab64613), calcitonin gene-related peptide (mouse, 1:200, Abcam, Cat. No. ab81887), Neurofilament 200 (mouse, 1:200, Abcam, Cat. No. ab215903). For visualization, IB_4_-fluorescein isothiocyanate (FITC) (1:200, Sigma‒Aldrich; Cat. No. L2895) or appropriate secondary antibodies, including Alexa Fluor 555 goat anti-rabbit (1:300, Cell Signaling Technology, Cat. No. #4413) and Alexa Fluor 488 goat anti-mouse (1:300, Cell Signaling Technology, Cat. No. #4408), were utilized. Fluorescence images were captured using a Nikon104c microscope equipped with a CoolSnap-ProColor CCD camera (Photometrics).

### Measurement of cAMP levels

The measurement of cAMP contents was carried out as described previously [28]. Lysates from mouse TG cells were prepared with proper amounts of 0.1 M HCL for 10 min. Samples were homogenized and centrifuged at a speed of 600 g for 5 min at room temperature. Supernatants were used for cAMP assays, using a cAMP immunoassay kit according to the manufacturer’s directions (BBI, Shanghai).

### TG microinjection

Intra-TG injection was administered with a 22-gauge needle as described previously [19, 25, 26]. The needle tip was positioned at the medial section of the TG, and drugs or reagents were administrated slowly in a volume of 3 µl over a period of 5 minutes. Chemical modified small interfering RNA (siRNA) targeting IL-20R1 (5’- CUAUGGUCAUUAUGAGAGATT-3’), IL-22R1 (5’-CUCCGAUAUUGUCCAAGGATT-3’), Lyn (5’-GCGAGAGUCAUCGAAGAUATT-3’), Cav3.2 (5’-GCUUGGGAACGUGCUUCU UTT-3’) as well as scrambled negative control siRNAs were obtained from RiboBio Tech. The siRNAs, tagged with 6-carboxyfuorescein (6-FAM), had the 5’-Cholesteryl- and 2’-O-methyl-modifications to respectively increase the stability and permeability, and were administered daily for two consecutive days.

### Animal model and behavioral studies

Mice were maintained on a circadian 12-h light/12-h dark cycle with food and water available *ad libitum*. All efforts were made to reduce the number of animals used and to minimize their suffering during procedures. Chronic constriction injury to the infraorbital nerve (CCI-ION) was chosen to establish a trigeminal neuralgia model in mice, following the protocol described in our previous studies [19, 26]. Briefly, mice were anesthetized with isoflurane, and the left infraorbital nerve was carefully separated near the infraorbital foramen. Two loose silk ligatures (4/0) were placed (with 1–2 mm spacing) around the ION. The sham-treated group underwent a similar procedure without ligation. The tests were performed in a blind manner that the investigator is not aware of the identification of animals as well as the study groups. The escape threshold to mechanical stimulation was assessed using *von* Frey filaments provided by Ugo Basile, with bending forces ranging from 0.008 to 2 g [20, 25]. The stimuli were gently applied to the skin within the infraorbital nerve territory, close to the center of the vibrissal pad. Each stimulation was repeated three times within each trial session, with a minimum 1-minute interval between each, and lasting for 2 to 3 s. These stimulations aimed to elicit behavior responses such as head withdrawal or escape, aligning with the approach introduced by Vos et al. [29].

### Pharmacological reagents

Chemicals were purchased from Sigma-Aldrich unless otherwise indicated. IL-24, Rp-cAMPs, and SB203580 were obtained from MedChemExpress. GPLG0634 and Bafetinib were purchased from Apexbio. GF109203X, Go6976, and PKI 6–22 were purchased from Tocris. TTA-P2 was obtained from Alomone Labs. Stock solutions of these compounds were prepared by dissolving them in less than 0.05% dimethylsulfoxide (DMSO) and did not have significant effects on T-currents.

### Statistical analysis

Data are expressed as means ± S.E.M. Prism 8.0, Clampft 10.2, and Microsoft Excel were utilized for data gathering and analysis. Currents from before and after drug application were compared using a paired t-test, and an unpaired t test was examined to compare two independent groups. ANOVA with Bonferroni post hoc tests were used for multiple comparisons. The behavioral data were analyzed using two-way repeated-measures (RM) ANOVA followed by Bonferroni post hoc test. Differences with *p* < 0.05 were considered statistically significant. The concentration-response curve data for IL-24 were fitted using the Hill equation: I/I_control_= 1/(1 + 10(log EC_50_ − X)*n*), where *n* is the Hill coefficient, X is the decadic logarithm of the agonist concentration, and EC_50_ is the concentration of agonist that elicits a 50% maximum response. The voltage-dependent activation data were fitted by the following modified Boltzmann equation: G/G_max_=1/{1 + exp[–(*V*_1/2_–*V*_m_)/*k*]}, where G_max_ is the fitted maximal conductance, *V*_1/2_ is the membrane potential for half-activation, and *k* is the slope factor. Steady-state inactivation data were fitted with the following negative Boltzmann equation: I/I_max_=1/{1 + exp[-(*V*_1/2_–*V*_m_)/*k*]}, where I_max_ is maximal current.

## Results

### IL-24 enhances T-currents in TG neurons

**Fig. 1:**
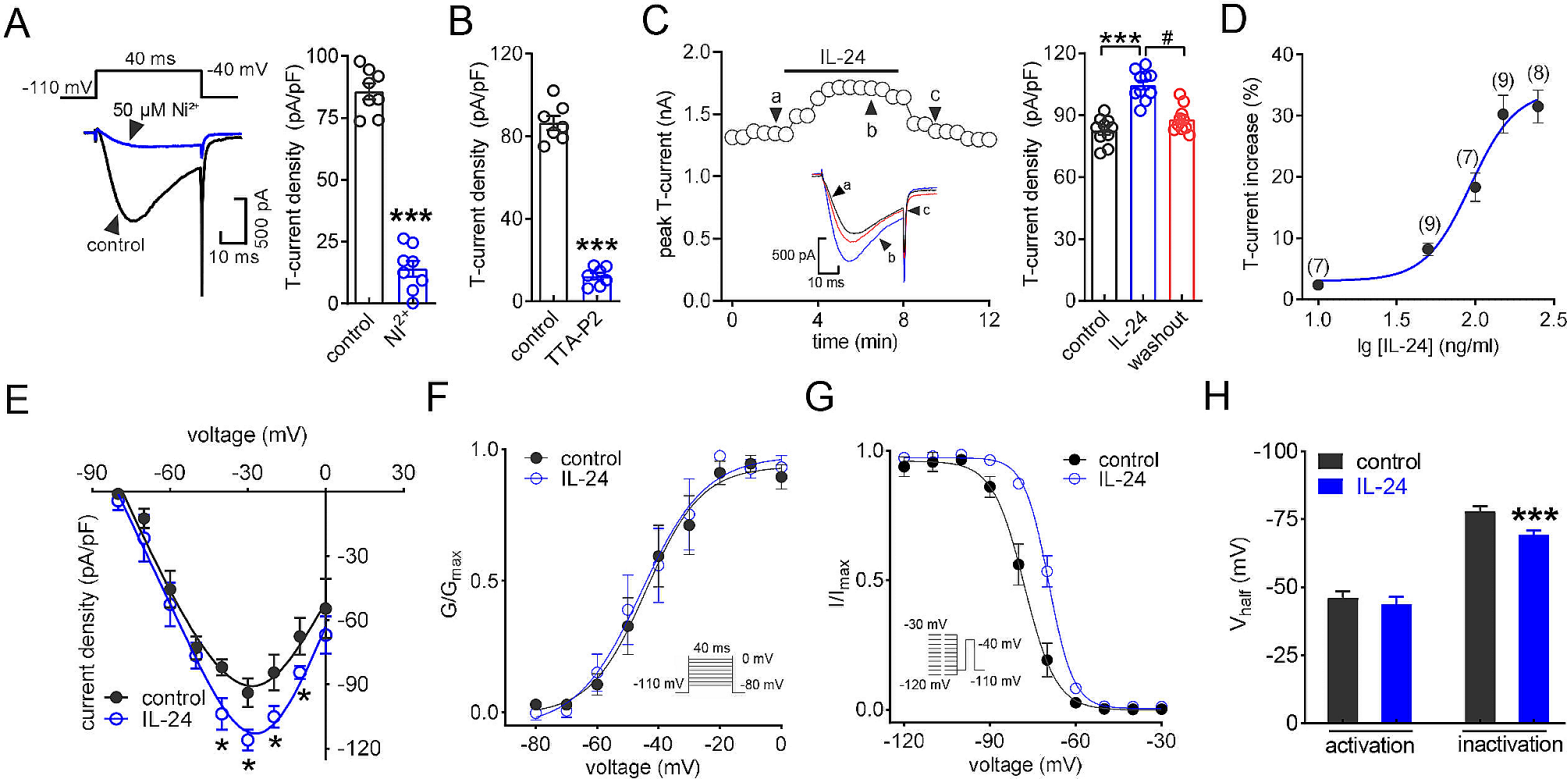
IL-24 enhances T-currents in small-sized TG neurons. (**A**) The left panel shows representative traces, and the right panel provides a summary data, indicating the inhibition of T-currents by Ni^2+^ (50 µM) (*n* = 8 cells). ***p < 0.001 (compared to control, paired t-test). (**B**) Summary data revealing the effect of TTA-P2 (3 µM) on T-currents in TG neurons (*n* = 7 cells). ***p < 0.001 (compared to control, paired t-test). (**C**) Time course of current changes (*left panel*) and summary data (*right panel*) demonstrate the enhancement of T-currents induced by 150 ng/ml IL-24 (*n* = 10 cells). ***p < 0.001 (compared to control, paired t-test). ^#^*p* < 0.05 (compared to IL-24, paired *t*-test). (**D**) A dose-response curve of IL-24 on T-currents is depicted, with the solid line representing the best fit using the Hill equation. The number of cells recorded at each IL-24 dose is displayed in round brackets. (**E**) The current-voltage curve displays the increased T-current density induced by 150 ng/ml IL-24 (*n* = 12 cells). *p < 0.05 (compared to control, one-way ANOVA). (**F-G**) IL-24 at 150 ng/ml did not alter the voltage-dependent activation properties of T-type channels (**F**, *n* = 12 cells) but shifted steady-state inactivation properties in a depolarizing direction (**G**, *n* = 12 cells). *Insets*, simulation waveforms. (**H**) Summary of results of *V*_half_. ***p < 0.001 (compared to control, one-way ANOVA)

In the current study, we restricted our electrophysiological recording to small-sized TG neurons as they play essential roles in the nociceptive signaling [26, 30]. To isolate T-currents, we applied a cocktail of channel blockers in the bath solution which contains nifedipine (5 µM) to block L-type channels, ω-conotoxin MVIIC (0.2 µM) to block P/Q- and N-type channels, and SNX482 (0.2 µM) to block R-type channels. Currents were recorded by maintaining the neuron at -110 mV and applying a step to -40 mV. As shown in Fig. 1A and B, the peak amplitude of T-currents was dramatically decreased by 50 µM NiCl_2_ (decrease of 86.1 ± 7.2%; Fig. 1A) or 3 µM TTA-P2 (decrease of 91.7 ± 2.8%; Fig. 1B), demonstrating the effectiveness of T-current isolation. Further, the application of 150 ng/ml IL-24 to small-sized TG neurons resulted in a significant increase in the peak amplitude of T-currents (increase of 30.2 ± 2.7%) and the increase induced by IL-24 was partially recovered after washout (Fig. 1C). Further investigations into the IL-24 response revealed that IL-24 augmented T-currents in a manner dependent on the dosage. The association between IL-24 concentration and the extent of augmentation was characterized by a sigmoidal Hill equation, with a median effective concentration (EC_50_) of 93.2 ng/ml (Fig. 1D). Furthermore, we explored the biophysical properties underlying IL-24-mediated T-current increase. We found that the application of 150 ng/ml IL-24 caused a downward shift in the current-voltage relationship curve (Fig. 1E). In addition, our findings showed that application of IL-24 had no significant effect on the voltage of half-maximal activation (Fig. 1F and H), but induced an ∼ 8.5 mV depolarizing shift in steady-state inactivation (Fig. 1G and H), implying that the reduced proportion of T-type channels in a state of steady-state inactivation may contribute to the IL-24-induced increase in T-currents.

### IL-22R1 mediates the IL-24induced T-type channel response

**Fig. 2:**
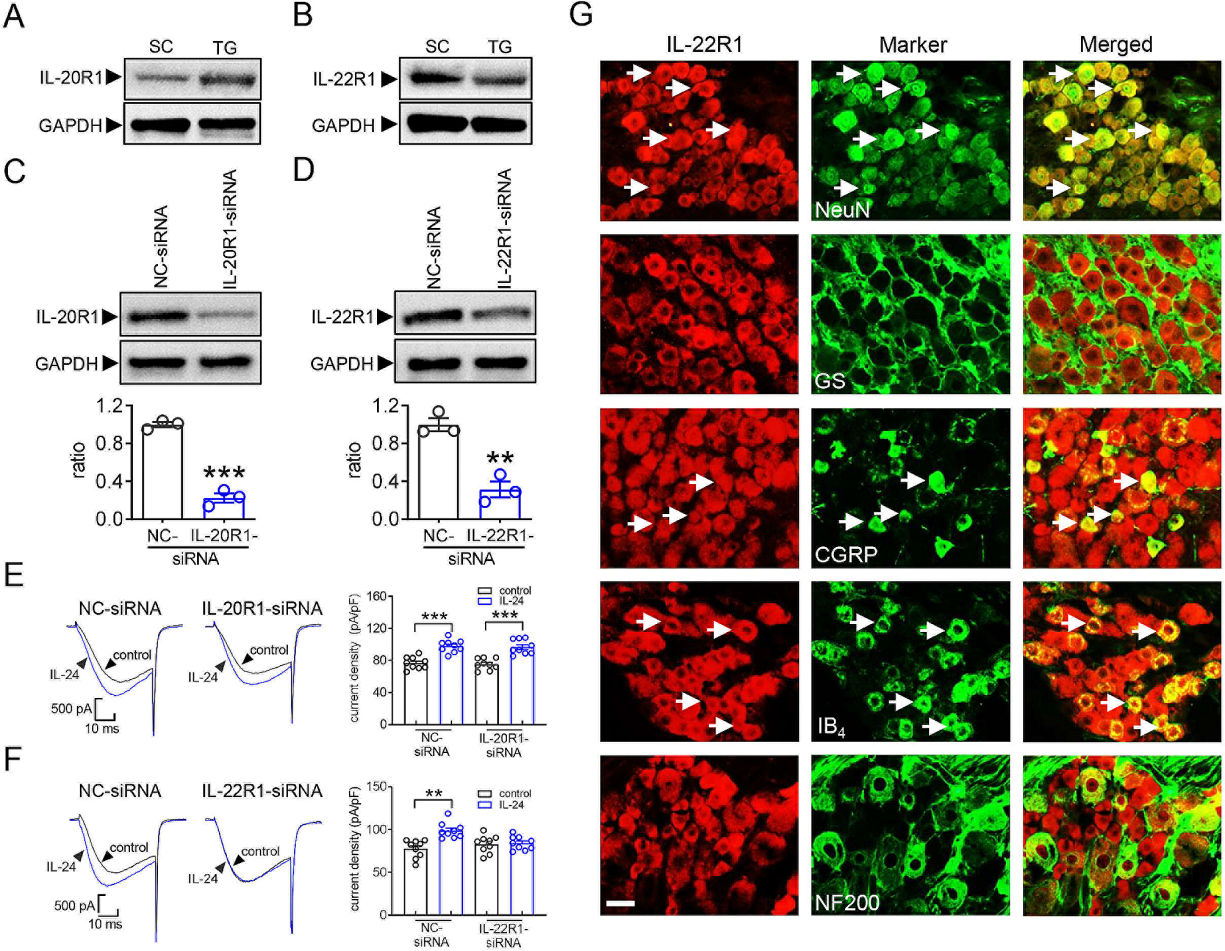
IL-22R1 mediates the IL-24-induced increase in T-currents. (**A-B**) Immunoblot analysis of IL-20R1 (*A*) and IL-22R1 (*B*) in the spinal cord (SC) and TG of intact mice. Representative blots from at least 3 independent experiments are displayed. (**C-D**) Protein abundance of IL-20R1 (*C*) or IL-22R1 (*D*) in TG cells treated with IL-20R1 siRNA (IL-20R1-siRNA) or IL-22R1-siRNA. NC-siRNA, negative control siRNA. **p < 0.01 and ***p < 0.001 (compared to NC-siRNA, unpaired *t-*test). Representative blots from at least 3 independent experiments are displayed. (**E-F**) Representative traces (*left*) and summary data (*right*) indicating that treatment of IL-22R1-siRNA (*n* = 9 cells), but not IL-20R1-siRNA (*n* = 9 cells), prevented the IL-24-induced T-current increase. IL-24 at 150 ng/ml significantly enhanced T-currents in TG cells transduced with NC-siRNA in panels E (*n* = 9 cells) and F (*n* = 9 cells), respectively. **p < 0.01 and ***p < 0.001 (compared to control, unpaired *t-*test). (**G**) Colocalization of IL-22R1 (*red*) with NeuN, GS, CGRP, IB_4_ or NF200 (*green*) in TG sections of intact mice. White arrows indicate colocalization. Scale bar, 50 μm

In mammals, the IL-20R1/IL-20R2 and IL-22R1/IL-20R2 heterodimeric receptors have been identified as the functional receptors responsible for IL-24 [2, 31]. Therefore, we determined the exact receptor involved in the IL-24-mediated T-current increase. Immunoblotting analysis of mouse TG lysates revealed that both IL-20R1 (Fig. 2A & Fig. S1) and IL-22R1 (Fig. 2B & Fig. S2) were endogenously expressed. Similar bands were also found in the spinal cord of dorsal horn tissues (Fig. 2A and B). To further examine the role of these receptors in the IL-24-mediated T-current response, a siRNA-mediated knockdown approach using chemically modified (2’-O-methyl- and 5’-cholesteryl-modified) IL-20R1-siRNA or IL-22R1-siRNA was adopted due to the commercial unavailability of specific inhibitors for IL-20R1 or IL-22R1. When compared to the corresponding control siRNA (NC-siRNA), intra-TG administration of either IL-20R1-siRNA (Fig. 2C & Fig. S3) or IL-22R1-siRNA (Fig. 2D & Fig. S4) respectively resulted in a significant decrease in the protein abundance of IL-20R1 or IL-22R1. IL-24 at 150 ng/ml enhanced T-currents in either IL-20R1-siRNA- or its negative control (NC-siRNA)-treated TG neurons (Fig. 2E), while knockdown of IL-22R1 completely abolished the IL-24–induced T-current response (increase of 2.9 ± 0.8%; Fig. 2F). Notably, 150 ng/ml IL-24 still robustly increased T-currents in neurons treated with the negative control of IL-22R1-siRNA (increase of 30.6 ± 4.3%; Fig. 2F). These findings indicate that IL-24 modulates T-currents by targeting the IL-22R1 receptor. To support this hypothesis, immunostaining analysis of TG tissue sections demonstrated that IL-22R1 expression was colocalized with the neuronal marker NeuN, while it was not observed in glutamine synthetase (GS)-labeled satellite glial cells (Fig. 2G). Additional differentiation of different types of neurons, including small unmyelinated peptidergic or nonpeptidergic neurons as well as medium and large myelinated neurons, using various phenotypic markers such as calcitonin gene-related peptide (CGRP), isolectin B4 (IB4), and 200 kDa neurofilament protein (NF200), revealed that IL-22R1 expression overlapped with IB4 and CGRP, but exhibited limited colocalization with NF200 (Fig. 2G).

### Lyn is required in the IL-24/IL-22R1-induced T-current increase

**Fig. 3:**
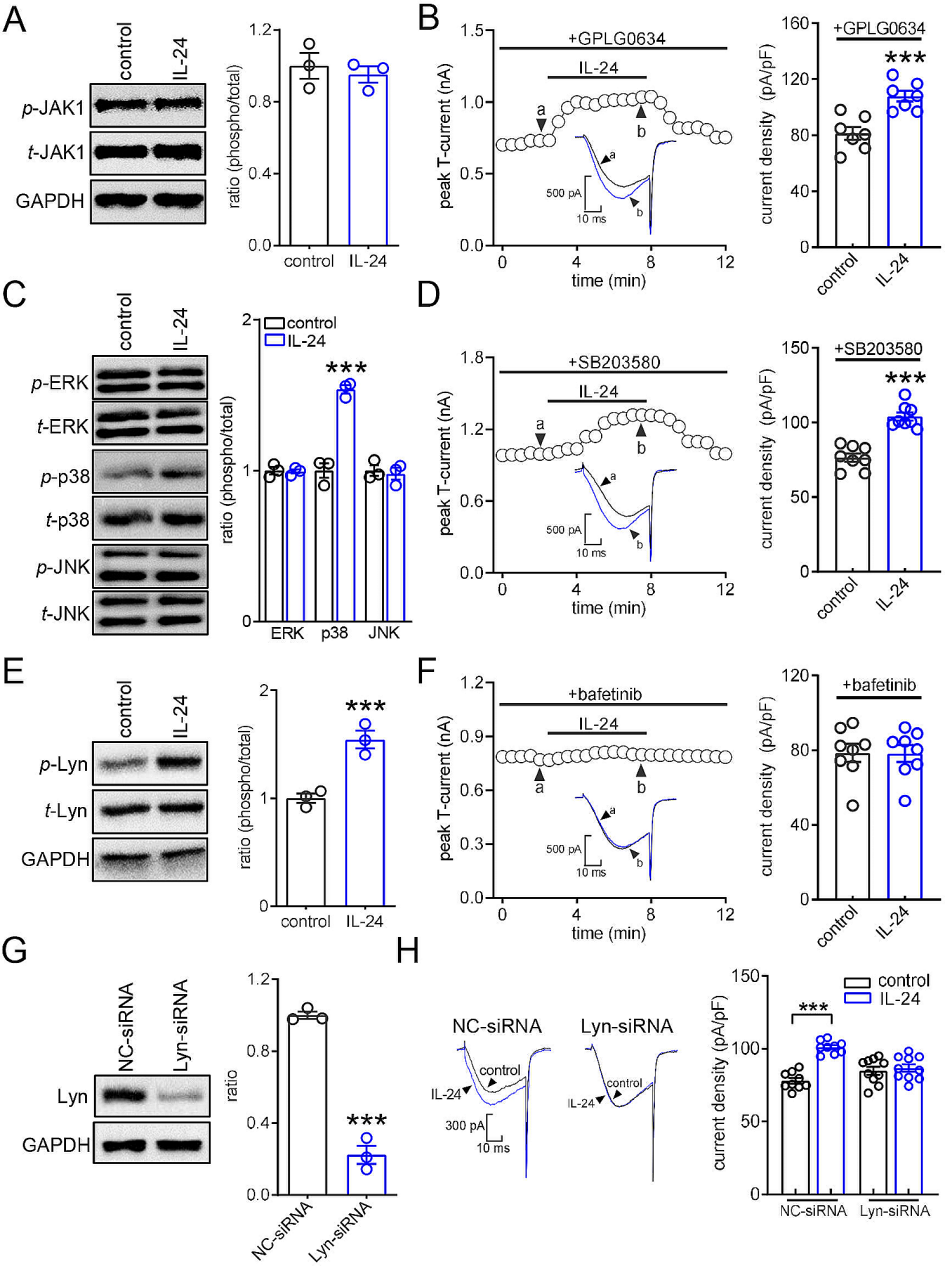
The IL-22R1-induced T-current response required the protein kinase Lyn. (**A**) The protein abundance of phosphorylated JAK1 (*p*-JAK1) or total JAK1 (*t*-JAK1) in TG cells treated with 150 ng/ml IL-24. Representative blots from at least 3 independent experiments are displayed. (**B**) The left panel shows representative traces, and the right panel provides a summary data, indicating the effect of IL-24 (150 ng/ml) on T-currents in cells pretreated with GPLG0634 (1 µM) (*n* = 7 cells). Letters (**a** and **b**) denote the points used for exemplary current traces. ***p < 0.001 (compared to control, paired *t-*test). (**C**) IL-24 increased the protein levels of phosphorylated p38 (*p*-p38), with no significant changes in *p*-ERK and *p*-JNK. Representative blots from at least 3 independent experiments are displayed. ***p < 0.001 (compared to control, unpaired *t-*test). (**D**) The left panel shows representative traces, and the right panel provides a summary data, indicating the effect of IL-24 (150 ng/ml) on T-currents in cells pretreated with SB203580 (10 µM) (*n* = 8 cells). Letters (**a** and **b**) denote the points used for exemplary current traces. ***p < 0.001 (compared to control, paired *t-*test). (**E**) Immunoblot analysis of *p*-Lyn or *t*-Lyn in TG cells treated with 150 ng/ml IL-24. Representative blots from at least 3 independent experiments are displayed. ***p < 0.001 (compared to control, paired *t-*test). (**F**) The left panel shows representative traces, and the right panel provides a summary data, indicating the effect of IL-24 (150 ng/ml) on T-currents in cells pretreated with bafetinib (1 µM) (*n* = 8 cells). Letters (**a** and **b**) represent points used for exemplary current traces. (**G**) Protein abundance of Lyn in TG cells treated with Lyn-siRNA. ***p < 0.001 (compared to NC-siRNA, unpaired *t-*test). Representative blots from at least 3 independent experiments are displayed. (**H**) Representative traces and bar graph indicating that treatment of Lyn-siRNA (*n* = 10 cells), but not NC-siRNA (*n* = 9 cells), prevented the IL-24-induced T-current increase. IL-24 at 150 ng/ml significantly enhanced T-currents in TG cells transduced with NC-siRNA in panel H (*n* = 9 cells). ***p < 0.001 (compared to control, unpaired *t-*test)

Previous studies have shown that IL-22R1-induced biological responses typically involve JAK1-mediated signaling [32]. Therefore, we investigated whether IL-24-mediated T-current responses were dependent on JAK1. However, in mouse TG cells, the levels of total JAK1 (*t*-JAK1) and phosphorylated JAK1 (*p*-JAK1) were not affected by IL-24 at a concentration of 150 ng/mL (Fig. 3A & Fig. S5). Similarly, pretreating TG neurons with the JAK1-specific inhibitor GPLG0634 did not alter the stimulatory effect of IL-24 on T-currents (increase of 31.1% ± 4.7%; Fig. 3B). Additionally, MAPK signaling, known to mediate IL-22R1 downstream signaling and play a crucial role in pain regulation [33], was examined for its involvement in IL-24/IL-22R1-induced T-current increase in TG neurons. Immunoblotting analysis revealed that exposure of TG cells to 150 ng/mL IL-24 significantly increased the expression of phosphorylated p38 (*p*-p38), while the levels of total p38 (*t*-p38), phosphorylated ERK (*p*-ERK), and phosphorylated JNK (*p*-JNK) remained unchanged (Fig. 3C & Fig. S6), suggesting a potential involvement of p38-mediated signaling in IL-24-induced biological responses. Surprisingly, pretreatment with a selective p38 MAPK inhibitor, SB203580, did not affect the IL-24-induced T-current increase (increase of 32.9 ± 2.5%; Fig. 3D). Moreover, we examined whether the activation of Lyn, a nonreceptor tyrosine kinase involved in various cytokine signaling pathways [34], was necessary for IL-24 action in TG neurons. Exposure of TG cells to 150 ng/mL IL-24 significantly increased the phosphorylation of Lyn (*p*-Lyn), while the total Lyn (*t*-Lyn) level remained unchanged (Fig. 3E & Fig. S7). Further, when TG neurons were pretreated with the small-molecule Lyn inhibitor bafetinib (1 µM), the IL-24-induced increase in T-currents was completely abolished (increase of 2.3 ± 1.7%; Fig. 3F). To further examine the role of Lyn in the IL-24-mediated T-current response, a siRNA-mediated knockdown approach using chemically modified Lyn-siRNA was applied. When compared to the corresponding control siRNA (NC-siRNA), intra-TG administration of Lyn-siRNA resulted in a significant decrease in the protein abundance of Lyn (Fig. 3G & Fig. S8). IL-24 at 150 ng/ml enhanced T-currents in NC-siRNA-treated TG neurons (Fig. 3H), while knockdown of Lyn completely abolished the IL-24–induced T-current response (increase of 3.5 ± 1.2%; Fig. 3H). These findings demonstrate that Lyn activation is necessary for the IL-24/IL-22R1-induced response in T-type channel activity.

### The IL-24-induced T-type channel response requires cAMP-independent PKA

**Fig. 4:**
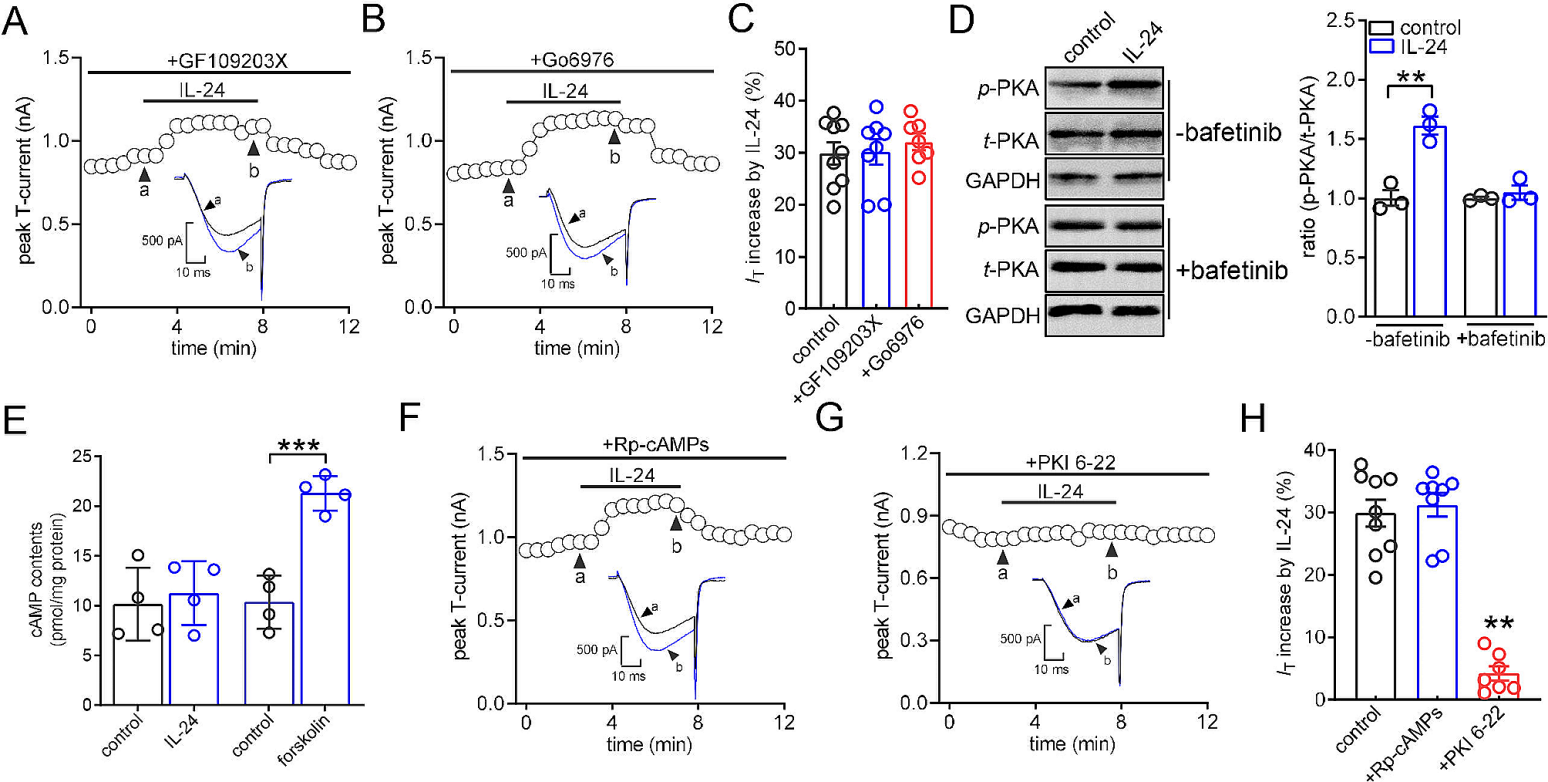
PKA is involved in the IL-24-mediated T-current response. (**A-B**) Time course of T-current changes demonstrating the effect of IL-24 (150 ng/ml) on T-currents in TG cells preincubated with 1 µM GF109203X (*A*) or 1 µM Go6976 (*B*), respectively. Letters (**a** and **b**) represent points used for exemplary current traces. (**C**) Summary of results revealing the effect of IL-24 (150 ng/ml) on T-currents in TG neurons pretreated with GF109203X (*n* = 8 cells) or Go6976 (*n* = 7 cells). (**D**) Protein expression of phosphorylated PKA (*p*-PKA, PKA activation) in TG cells induced by IL-24 (150 ng/ml) in the presence or absence of bafetinib (1 µM). Representative blots from at least 3 independent experiments are displayed. **p < 0.01 (compared to control, unpaired *t-*test). (**E**) Application of forskolin (20 µM), but not IL-24 (150 ng/ml), increased cAMP contents in TG cells. ***p < 0.001 (compared to control, unpaired *t-*test). (**F-G**) Time course of T-current changes demonstrating the effect of IL-24 (150 ng/ml) on T-currents in TG neurons pretreated with Rp-cAMPs (10 µM) (*F*) or dialyzed with PKI 6–22 (5 µM) (*G*), respectively. Letters (**a** and **b**) denote the points used for exemplary current traces. (**H**) Bar graph showing the effect of 150 ng/ml IL-24 on T-currents in cells pretreated with Rp-cAMPs (*n* = 8 cells) or dialyzed with PKI 6–22 (*n* = 7 cells). **p < 0.01 (compared to control, unpaired *t*-test)

Evidence has suggested the involvement of protein kinase C (PKC) in the regulation of T-currents [35, 36]. However, pretreating TG neurons with GF109203 × (1 µM), a PKC inhibitor, did not affect the ability of IL-24 to increase T-currents (increase of 30.1 ± 3.9%; Fig. 4A and C). Similar results were obtained with another PKC inhibitor, Go6976 (200 nM) (increase of 32.1 ± 2.2%; Fig. 4B and C). Research findings have indicated the involvement of PKA-dependent regulation in T-type channels [37, 38]. Western blot analysis showed that exposure of TG cells to IL-24 (150 ng/ml) led to a significant increase in the phosphorylation of PKA (p-PKA, indicative of PKA activation). Importantly, pre-treatment with the Lyn inhibitor bafetinib (1 µM) abolished the IL-24-induced PKA activation (Fig. 4D & Fig. S9), suggesting the involvement of PKA in IL-24-mediated Lyn signaling. PKA is an important effector enzyme commonly activated by cAMP [39]; thus, we assessed cAMP contents in TGs and surprisingly found that cAMP contents in TGs were unchanged after IL-24 administration (Fig. 4E). Contrastingly, application of the cAMP elevating agent forskolin (20 µM) dramatically increased cAMP contents in TG cells (Fig. 4E). Further pretreating TG neurons with Rp-cAMPs (10 µM), a membrane-permeable competitive cAMP antagonist, did not affect the IL-24-mediated T-current response (increase of 31.7 ± 3.9%; Fig. 4F and H). However, dialyzing small-sized neurons with PKI 6–22 (5 µM), a PKA peptide inhibitor but not a cAMP inhibitor, abrogated the IL-24-induced T-current increase (increase of 4.2 ± 2.1%; Fig. 4G and H). These results demonstrated that IL-24 stimulated T-type channels through PKA, but independent of cAMP.

### IL-24 increases TG neuronal excitability

**Fig. 5:**
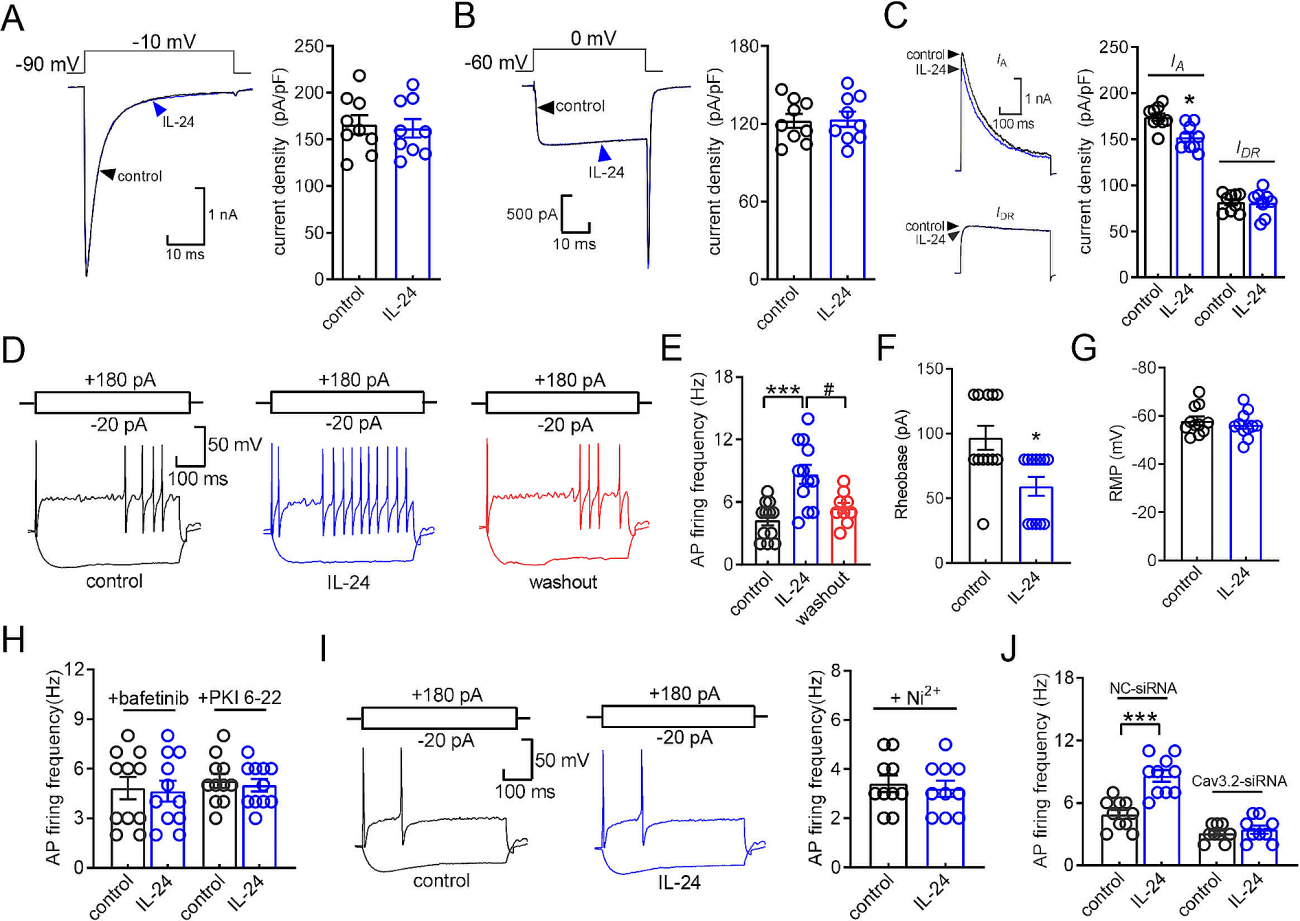
IL-24 induces TG neuronal hyperexcitability. (**A-C**) Representative traces (*left*) and summary of results (*right*) demonstrating the effect of IL-24 (150 ng/ml) on Nav currents (*A*, *I*_Na_, *n* = 9 cells), high-voltage activated (HVA) Ca^2+^ currents (*B*, *n* = 9 cells), transient outward K^+^ channel currents (*I*_A_, *upper panel*) (*C*, *n* = 9 cells), or sustained delayed-rectifier K^+^ currents (*I*_DR_, *lower panel*) (*C*, *n* = 9 cells). *I*_Na_ was elicited from the holding potential of -90 mV and depolarized to -10 mV. HVA Ca^2+^ currents were elicited from the holding potential of -60 mV and depolarized to 0 mV. Kv currents are depolarized from a holding potential of -80 mV to + 40 mV. To obtain *I*_A_, a 150 msec prepulse to -10 mV was included to inactivate the transient channels, resulting in sustained *I*_DR_ isolation. Offline subtraction of *I*_DR_ from the total current yielded *I*A. *p < 0.05 (compared to control, paired *t-*test). (**D-E**) Representative traces (*D*) and summary of results (*E*) demonstrating that IL-24 at 150 ng/ml significantly increased the action potential (AP) firing rate (*n* = 12 cells). *Insets* indicate the protocols of current injection. ***p < 0.001 (compared to control, paired *t-*test). ^#^*p* < 0.05 (compared to IL-24, paired t-test). (**F-G**) Effect of 150 ng/ml IL-24 on the rheobase (*F*) and resting membrane potential (RMP) (*G*) of AP firing (*n* = 12 cells). *p < 0.05 (compared to control, paired *t-*test). (**H**) Bar graph revealing that pretreatment of TG neurons with bafetinib (1 µM, *n* = 11 cells) or dialyzed with PKI 6–22 (5 µM, *n* = 11 cells) completely abolished the increased AP firing rate induced by IL-24. (**I**) The left panel shows representative traces, and the right panel provides a summary data, indicating that application of Ni^2+^ at 50 µM abrogated the increased AP firing rate induced by IL-24 (*n* = 10 cells). (**J**) Summary of results revealing that Cav3.2-siRNA (*n* = 9 cells), but not its NC-siRNA (*n* = 10 cells), prevented the 150 ng/ml IL-24-induced increase in AP firing rate. ***p < 0.001 (compared to control + NC-siRNA, unpaired *t-*test)

To investigate the functional roles of IL-22R1-mediated modulation of T-type channels, we conducted further experiments to determine whether IL-24 affects the membrane excitability of TG neurons. Initial examination showed that the application of 150 ng/mL IL-24 had no effect on voltage-gated Na^+^ currents (decrease of 2.9 ± 1.2%; Fig. 5A). Given previous indications of PKA’s role in enhancing cardiac Cav1.2 L-type Ca^2+^ channels [40], we further investigated by applying 5 µM nifedipine to block L-type channels. Interestingly, our findings showed that IL-24 at 150 ng/mL did not significantly influence the remaining high voltage-activated Ca^2+^ currents (increase of 1.7 ± 0.6%; Fig. 5B). Moreover, application of IL-24 was found to reduce the peak amplitude of transient outward A-type K^+^ currents (*I*_A_) by 17.3 ± 3.5% (Fig. 5C), while delayed rectifier K^+^ currents (*I*_DR_) remained unaffected (increase of 3.5 ± 0.9%; Fig. 5C). As a result, in the presence of an extracellular solution containing 5 µM nifedipine to block L-type channels and 5 mM 4-aminopyridine (4-AP) to block *I*_A_ channels, the application of IL-24 at a concentration of 150 ng/ml significantly increased the rate of action potential (AP) firing by 91.6 ± 5.9% (Fig. 5D and E). IL-24-treated TG neurons exhibited a lower rheobase compared to non-treated neurons (Fig. 5F). However, Other characteristics including the resting membrane potential (Fig. 5G) remained unchanged. In addition, pretreating TG neurons with the Lyn inhibitor bafetinib (1 µM) completely eliminated the IL-24-induced increase in AP firing rate (Fig. 5H). Similar results were observed when TG neurons were dialyzed with PKI 6–22 (5 µM; Fig. 5H). To determine if the TG neuronal hyperexcitability caused by IL-24 is dependent on T-type channel stimulation, cells were pretreated with Ni^2+^ (50 µM), which resulted in the abolishment of the IL-24-induced increase in AP firing rate (Fig. 5I). Furthermore, we utilized a chemically modified siRNA knockdown approach to examine the participation of Cav3.2, the primary subtype of T-type channels in peripheral sensory neurons, in IL-24-induced neuronal hyperexcitability. Intra-TG injection of Cav3.2-siRNA significantly reduced the protein expression of Cav3.2 (Fig. S10). The IL-24-induced increase in the AP firing rate was completely abolished when the expression of Cav3.2 was knockdown using Cav3.2-siRNA (increase of 3.1 ± 1.8%; Fig. 5J). In contrast, in the NC-siRNA-treated mice, the increase remained unaffected (increase of 87.5 ± 7.6%; Fig. 5J).

### Involvement of T-type channels in IL-24-induced pain hypersensitivity

**Fig. 6:**
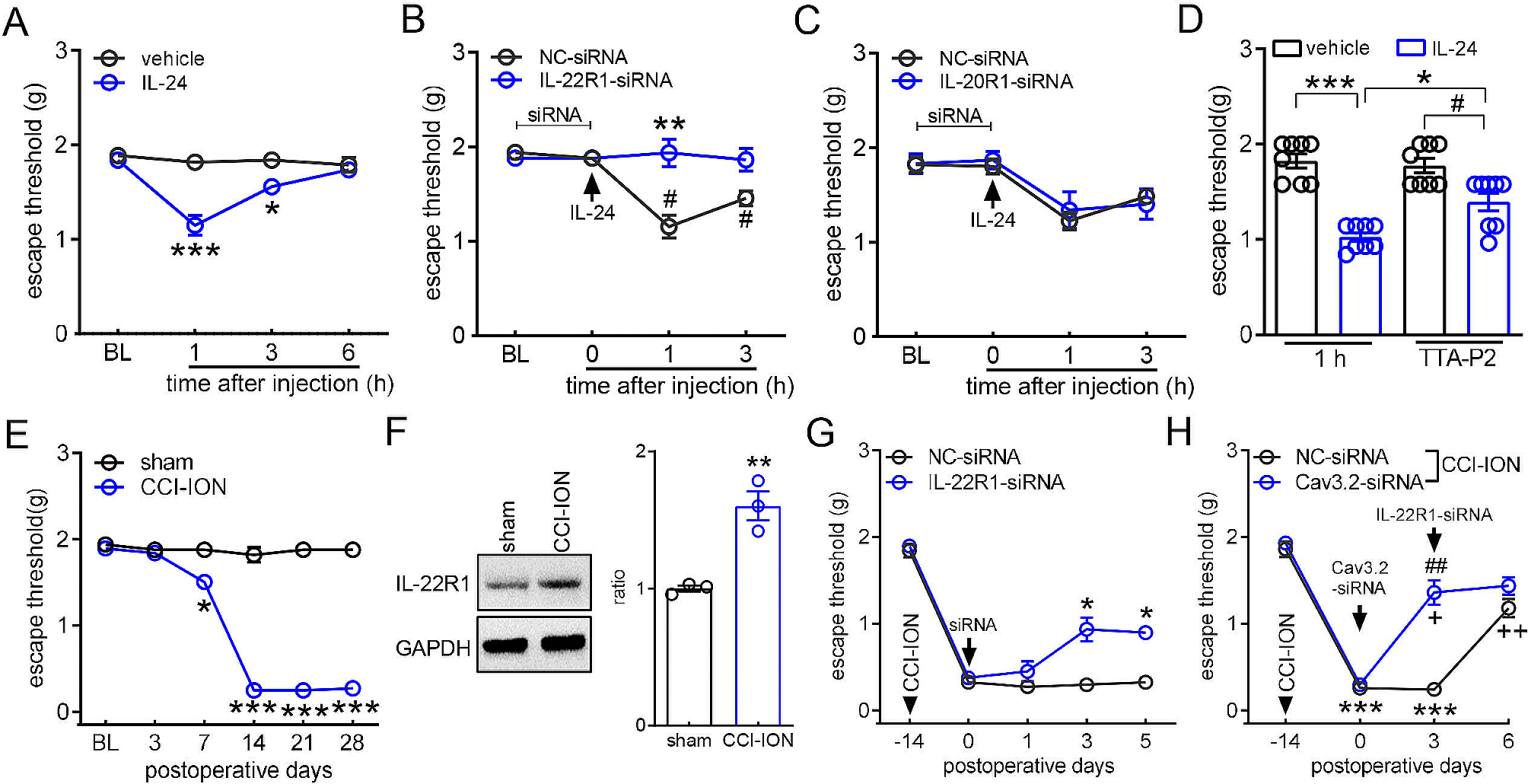
Peripheral IL-24/IL-22R1 induces mechanical pain hypersensitivity. (**A**) Escape threshold after intra-TG administration of 100 ng IL-24 or vehicle. *p < 0.05 and ***p < 0.001 (compared to vehicle, two-way ANOVA). BL, baseline. (**B-C**) Effects of IL-22R1-siRNA (*B*) or IL-20R1-siRNA (*C*) as well as the corresponding control siRNAs (NC-siRNAs) on IL-24 (100 ng, intra-TG injection; arrow)-induced mechanical hypersensitivity. **p < 0.05 (compared to NC-siRNA at 1 h) and ^#^*p* < 0.05 (compared to NC-siRNA at 0 h) (two-way ANOVA). (**D**) Pretreatment with TTA-P2 (1 nmol) attenuated 100 ng IL-24-induced mechanical hypersensitivity. *p < 0.05 and ***p < 0.001 (compared to IL-24 at 1 h), ^#^*p* < 0.05 (compared to vehicle + TTA-P2) (two-way ANOVA). (**E**) Escape threshold to mechanical stimuli in the sham- or CCI-ION-operated mice. *p < 0.05 and ***p < 0.001 (compared to sham, two-way ANOVA). (**F**) Immunoblot analysis of IL-22R1 in TGs on day 14 after CCI-ION-operation or sham surgery. **p < 0.01 (compared to sham, unpaired *t-*test). Representative blots from at least 3 independent experiments are displayed. (**G**) Intra-TG injection of IL-22R1-siRNA 14 days after CCI-ION alleviated mechanical pain hypersensitivity in CCI-ION mice. *p < 0.05 (compared to NC-siRNA at the corresponding time point, two-way ANOVA). (**H**) The effect of Cav3.2-siRNA versus NC-siRNA (Day 0) on IL-22R1-siRNA (Day 3)-induced alleviation of mechanical allodynia in CCI-ION mice. *** *p* < 0.001 (compared to CCI-ION at -14 days), ^+^*p* < 0.05 and ^++^*p* < 0.01 (compared to NC-siRNA at the 3-day point in CCI-ION mice), ^##^*p* < 0.01 (compared to Cav3.2-siRNA at the 0-day point in CCI-ION mice) (two-way ANOVA). For all animal behavior detection, *N* = 7–9 mice for each group

To gain further insight into the functional impact of IL-24 at the behavioral level, we sought to determine whether the application of IL-24 influences pain sensitivity in mice. We assessed the escape threshold by measuring the response to *von* Frey filaments for mechanical sensitivity. Following intra-TG injection of IL-24 (100 ng), a pronounced decrease in the escape threshold was observed, as shown in Fig. 6A. This effect was found to resolve 6 h after IL-24 administration. Moreover, the IL-24-induced mechanical pain hypersensitivity was counteracted by pretreatment with IL-22R1-siRNA delivered via intra-TG injection (Fig. 6B). In contrast, pre-injection of IL-20R1-siRNA failed to produce similar effects, as illustrated in Fig. 6C. Additionally, the involvement of T-type channels in IL-24-mediated mechanical hypersensitivity was further investigated using the specific T-type channel blocker, TTA-P2. While the intra-TG application of 1 nmol of TTA-P2 did not significantly impact the escape threshold, as shown in Fig. 6D, pretreatment with TTA-P2 notably mitigated the mechanical hypersensitivity induced by IL-24 (100 ng) (Fig. 6D). Additionally, we investigated the potential involvement of IL-24 in chronic neuropathic pain. A mouse model of trigeminal neuralgia was induced through chronic constriction injury to the infraorbital nerve (CCI-ION) [26, 41]. In comparison to sham surgery, mice displayed a significant decrease in escape threshold in response to mechanical stimuli on days 7, 14, 21, and 28 after the CCI-ION procedure (Fig. 6E). Notably, on Day 14, when mechanical allodynia reached its peak, the trigeminal ganglia (TGs) exhibited a robust concurrent increase in IL-22R1 protein levels (Fig. 6F & Fig. S11). Consequently, the influence of IL-22R1 inhibition on nerve injury-induced mechanical allodynia was further investigated. Intra-TG delivery of IL-22R1-siRNA led to a significant reduction in mechanical allodynia in CCI-ION-treated mice, in comparison to the NC-siRNA-treated groups (Fig. 6G). To validate the role of Cav3.2 channels as crucial targets for the pain hypersensitivity associated with IL-24 signaling in trigeminal neuropathic pain, we employed chemically modified Cav3.2-siRNA (or NC-siRNA) via intra-TG injection. The results demonstrated that the administration of Cav3.2-siRNA markedly alleviated mechanical allodynia in CCI-ION mice, whereas treatment with NC-siRNA did not improve the escape threshold (Fig. 6H). Subsequent assessment of mechanical sensitivity in Cav3.2-siRNA-treated CCI-ION mice following the injection of IL-22R1-siRNA revealed that IL-22R1-siRNA did not have an appreciably additional effect compared to Cav3.2-siRNA (Fig. 6H). In contrast, mice that received injections of NC-siRNA for Cav3.2 responded to IL-22R1-siRNA similarly to those that did not receive siRNA injection, as the administration of IL-22R1-siRNA resulted in a significant increase in the escape threshold (Fig. 6G and H). These findings imply that T-type channels, most likely Cav3.2 subtype, are involved in the IL-22R1-mediated hypersensitivity to pain in trigeminal neuralgia.

## Discussion

**Fig. 7:**
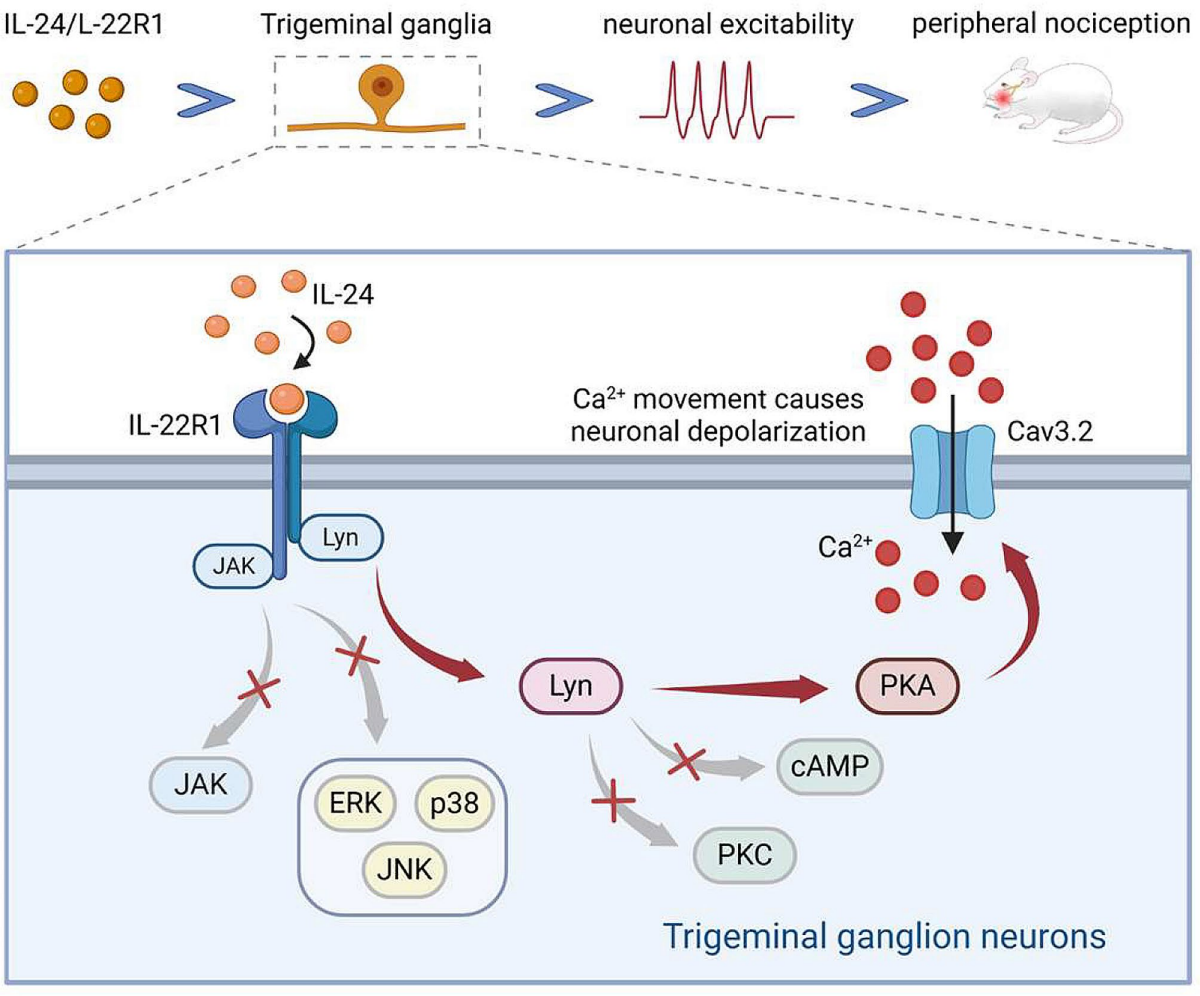
The proposed mechanisms of IL-22R1 signaling on T-type channels. IL-24 engages IL-22R1, leading to the activation of tyrosine protein kinase Lyn. Lyn then stimulates downstream cAMP-independent PKA, which in turn modulates the activity of T-type channels, resulting in an increase in T-currents. This signaling cascade mediated by IL-22R1 contributes to the hyperexcitability of trigeminal ganglion (TG) neurons and the nociceptive behaviors induced by IL-24. Notably, IL-22R1 is upregulated in the injured TG, and blocking Cav3.2 attenuates IL-22R1-mediated pain hypersensitivity in neuropathic pain behaviors induced by CCI-ION. JAK1, MAPK, and PKC do not appear to be involved in the IL-24-mediated response of T-currents. Created by Biorender.com

The present study shed light on the crucial role of IL-24 in regulating T-type channels in peripheral sensory neurons. Our results revealed that IL-24’s effects were facilitated through IL-22R1 binding to Lyn and initiating the activation of downstream PKA signaling (refer to Fig. 7 for an illustration of the proposed mechanism). The T-type channel response mediated by IL-22R1 contributes to heightened excitability in TG neurons and nociceptive behaviors. Targeting IL-22R1-mediated signaling may present a new therapeutic approach/ target for pain syndrome.

It has been demonstrated that IL-24 can function either as an intracellular cell death-inducing factor independent of receptor expression and JAK1/STAT signaling [42, 43], or as a classical cytokine through its cell surface receptors and downstream JAK1-dependent signaling [32]. In chicken nodose neurons, blockade of JAK inhibited the stimulatory effect of ciliary neurotrophic factor on T-type channels [44]. Similarly, trafficking of T-type channels required transient activation of the JAK signaling since stimulation with leukemia inhibitory factor evoked a considerable increase in the phosphorylation of JAK [45]. However, it is worth noting that in mouse TG neurons, the increase in T-type channels mediated by IL-24 was found to occur through a membrane receptor, independent of the JAK1 signaling cascade. This is supported by the fact that IL-24/IL-22R1 did not have an effect on the protein expression of p-JAK1, and the antagonism of JAK1 signaling did not prevent the T-type channel response induced by IL-24. Consistent with these findings, previous studies have shown that IL-24 induced apoptosis of melanoma cells through a novel receptor-mediated, but JAK1/STAT3-independent death pathway [46]. Both in vitro data and in *vivo* studies have established that ERK signalling plays a crucial role in nociceptive pain behaviours [47]. Ras-ERK signaling was identified to mediate the increased activity of T-type channels induced by Zinc transporter-1 [48], while in another study T-type channels were specifically inhibited in cells expressing oncogenically activated Ras as well as gain-of-function mutant MAPK/ ERK kinase [49]. In this study, it was observed that stimulation with IL-24 led to an elevation in the level of p-p38 in mouse TG cells, while p-ERK and p-JNK remained unaffected. This suggests that ERK is not involved in the IL-24-mediated T-current response. Furthermore, the possibility that the increase in T-currents induced by IL-24 was due to p38 activation can also be ruled out, as inhibition of p38 did not affect the IL-24-induced T-type channel response.

Protein kinase C (PKC) is considered a pivotal regulator of T-type channels [50]. Interestingly, studies examining the PKC-mediated modulation of T-type channels have conflicting conclusions, in that T-currents have been reported to be either upregulated or downregulated [50, 51]. For example, T-currents recorded from dorsal root ganglion (DRG) neurons were shown to increase in response to PKC activation induced by insulin-like growth factor [52]. A similar transduction cascade was revealed in genetically modified *Xenopus oocytes* expressing recombinant T-type channels [36]. However, the same effect could not be replicated in mammalian cells [38, 40, 53]. Contrastingly, in reticular thalamic neurons, PKC-mediated inhibition of T-type channels was identified [35]. Similarly, in DRG neurons, T-current suppression mediated by muscarinic M3 receptors was prevented by antagonism of PKC [54]. Thus, it appears that PKC differentially regulates the activity of T-type channels in a cell type- and signal-specific manner. Despite this, the involvement of PKC in the IL-22R1-mediated T-type channel response is unlikely, as inhibiting PKC did not impact the increase in IL-24-induced T-currents. Our results also indicate that Lyn-dependent PKA activity played a role in the IL-24-induced T-type channel response. This is supported by two key findings: (1) the Lyn inhibitor prevented IL-24-induced PKA activation, and (2) pre-treating TG neurons with either the PKA inhibitor KT-5720 or the Lyn inhibitor bafetinib eliminated the IL-24-induced T-current response. It’s worth noting that PKA is a significant effector enzyme commonly activated by cAMP. Interestingly in TG neurons of mice, IL-24/IL-22R1 stimulates T-currents via a cAMP-independent PKA signaling, since IL-24 application did not affect the content of cAMP in TG cells, and dialyzing small-sized neurons with PKI 6–22, a PKA peptide inhibitor but not a cAMP inhibitor, completely abolished the IL-24-induced T-type channel response. Consistently, pretreatment of TG neurons with the competitive cAMP antagonist Rp-cAMPs did not change the IL-24-induced T-current response. Indeed, these findings were supported by previous studies which have demonstrated the cAMP-independent PKA signaling in perivascular adipose tissue [55]. Additionally, studies have also demonstrated that PKA enhances T-currents in recombinant Cav3 channels [56]. Conversely, in retinal ganglion cells, the inhibition of T-currents by the cannabinoid CB1 and CB2 receptors was found to be blocked by PKA inhibitors [57]. Nevertheless, previous research has also revealed biphasic effects or even no impact of PKA activation on T-currents [38]. The heterogeneous effects of PKA on T-type channels indicate the involvement of different parameters in the modulation of T-type channel activity by PKA. The local microenvironment, influenced by tissue/cell-specific activation of endogenous PKA isoforms, contributes to the variability of PKA-dependent signal transduction pathways that affect ion channels [38, 50]. Additionally, the extensive alternative splicing isoforms of Cav3 subunits and relevant accessory subunits contribute to the diversity of PKA-mediated T-type channel activities [58–60]. It is worth noting that the three subunits of the mammalian Cav3 family (Cav3.1, Cav3.2, and Cav3.3) exhibit distinct properties and regulation [10, 61]. Parenthetically, it is important to consider the possibility that PKA phosphorylation by an intermediate protein may be involved in the observed IL-24-induced response, although this cannot be conclusively excluded.

T-type channels demonstrate a unique characteristic among voltage-gated Ca^2+^ channels by operating at the resting membrane potential of neurons [62]. This property makes them particularly important in peripheral nociceptors, as it leads to increased neurotransmission and heightened perception of pain [63, 64]. Recent studies have demonstrated that manipulating peripheral Cav3.2 channels can influence nociceptive inputs and that inhibiting T-type channels yields significant antinociceptive effects in various pain models [65, 66]. In our study, we provided evidence that IL-24 enhances excitability in trigeminal ganglion neurons, resulting in hypersensitivity to mechanical pain. These effects were prevented when IL-22R1 or Cav3.2 were knocked down using specific siRNAs. Moreover, blocking IL-22R1 signaling relieved mechanical allodynia in neuropathic pain induced by nerve injury, which was lessened by Cav3.2-siRNA administration. Thus, the stimulation of T-type channels by IL-24 played a role in the nociceptive effects caused by IL-22R1 activation. A similar connection has been proposed between IL-22R1 and the severity of chronic arthritis pain [67]. Further support of this finding is that spinal interleukin-24 contributes to neuropathic pain after peripheral nerve injury [6]. Importantly, recent evidence has reported that expression levels of both IL-24 and C-X-C motif chemokine ligand 13 (CXCL13) were upregulated in the DRG after nerve injury [68], although CCL11 and CXCL14 were comparatively lower. Interestingly, in another study, Zhao and colleagues have reported that the expression of CXCL13, but not IL-24, in synovial tissue of wrist arthritis was significantly higher than that in the normal control group [69]. These findings together suggested a differential regulation of expression of cytokines in distinct tissues and specific pathological conditions might exacerbate this difference. In addition, studies have shown that specific pathways in spinal microglia or DRG macrophages are implicated in the sexual dimorphism of neuropathic pain [70]. It appears that this dimorphism is primarily seen in microglial or macrophages, as inhibiting pain-related signaling in neurons and astrocytes leads to similar pain relief in both sexes [71]. Since IL-22R1 is exclusively expressed in TG neurons, it may account for the consistency of pain symptoms observed in male and female mice in the present study. Nonetheless, further investigation is still needed to fully understand the significance and underlying mechanism of how gender differences impact IL-22R1-mediated pain regulation.

## Conclusions

In summary, our research provides novel insights into the intricate molecular components involved in IL-24’s effects on T-type channels. Through our study, we present compelling evidence that stimulating IL-22R1 in TG neurons amplifies T-currents by activating Lyn-dependent, but cAMP-independent PKA signaling. This mechanism is believed to enhance neuronal excitability and contribute to the development of pain hypersensitivity. Importantly, our findings regarding the IL-22R1-mediated Lyn-PKA cascade in peripheral sensory neurons hold promising potential for the future development of targeted therapies in the field of clinical pain treatment.

### Electronic supplementary material

Below is the link to the electronic supplementary material.

Supplementary Material 1

## Data Availability

No datasets were generated or analysed during the current study.

## Abbreviations

IL-24: Interleukin-24
IL-22R1: Interleukin-22 receptor 1
TG: Trigeminal ganglion
T-currents: T-type Ca2 + channel currents
MAPK: Mitogen-activated protein kinase
ERK: Extracellular regulated protein kinases
*I*_A_: Transient outward A-type K^+^ currents
*I*_DR_: Delayed rectifier K^+^ currents
4-AP: 4-aminopyridine
CCI-ION: Chronic constriction injury to the infraorbital nerve
PKA: Protein kinase A
