# Supplementary material for: Interleukin-22 receptor 1-mediated stimulation of T-type Ca2+ channels enhances sensory neuronal excitability through the tyrosine-protein kinase Lyn-dependent PKA pathway
Source: Cell Commun Signal. 2024 Jun 3;22:307. doi: 10.1186/s12964-024-01688-6 (PMC11145867; doi:10.1186/s12964-024-01688-6)
Supplement: Supplementary file 1 — Supplementary Material 1 [file 12964_2024_1688_MOESM1_ESM.pdf]

Supporting Information

Interleukin-22 receptor 1-mediated stimulation of T-type Ca^{2+} channels enhances sensory neuronal excitability through the tyrosine-protein kinase Lyn-dependent PKA pathway

Hua Cai [a, #], Siyu Chen [a, b, #], Yufang Sun [b, c, #], Tingting Zheng [a], Yulu Liu [a], Jin Tao [b, c, d, *], Yuan Zhang [a, c, d, *]

[a] Clinical Research Center of Neurological Disease, Department of Geriatrics, The Second Affiliated Hospital of Soochow University, Suzhou 215004, P.R. China; [b] Department of Physiology and Neurobiology & Centre for Ion Channelopathy, Suzhou Medical College of Soochow University, Suzhou 215123, P.R. China; [c] Jiangsu Key Laboratory of Neuropsychiatric Diseases, Soochow University, Suzhou 215123, P.R. China; [d] MOE Key Laboratory of Geriatric Diseases and Immunology, Suzhou Medical College of Soochow University, Suzhou 215123, P.R. China.

[#] These authors contribute to this work equally.

Individual email addresses for all authors:

Hua Cai (caihua0224@163.com), Siyu Chen (20234233012@stu.suda.edu.cn), Yufang Sun (sunyf@suda.edu.cn), Tingting Zheng (shifinnztt@163.com), Yulu Liu (sdfeylyl@126.com), Jin Tao (taoj@suda.edu.cn), Yuan Zhang (yuanzhang@suda.edu.cn)

***To whom correspondence should be addressed:**

Dr. Yuan Zhang, Clinical Research Center of Neurological Disease, Department of Geriatrics, The Second Affiliated Hospital of Soochow University, Suzhou 215004, P.R. China. E-mail: yuanzhang@suda.edu.cn

Dr. Jin Tao, Centre for Ion Channelopathy, Department of Physiology and Neurobiology, Suzhou Medical College of Soochow University, Suzhou 215123, P.R. China. E-mail: taoj@suda.edu.cn

This PDF file includes: SI Figures S1 to S11

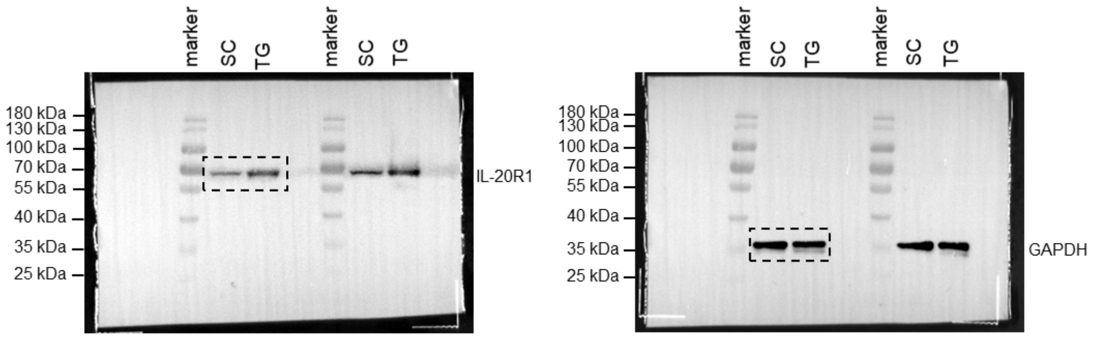

Fig. S1: Protein expression of IL-20R1 in the spinal cord (SC) and TG of intact mice. GAPDH was used as an equal loading control. Shown are the full-length images of immunoblots presented in Fig. 2A.

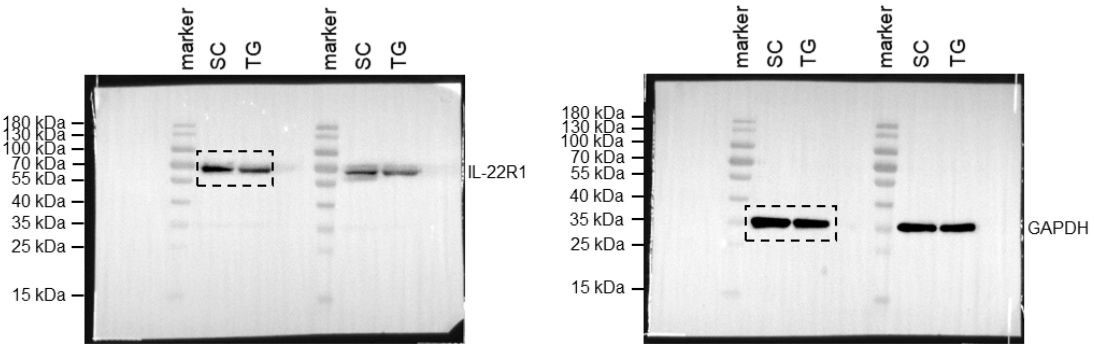

Fig. S2: Protein expression of IL-22R1 in the spinal cord (SC) and TG of intact mice. GAPDH was used as an equal loading control. Shown are the full-length images of immunoblots presented in Fig. 2B.

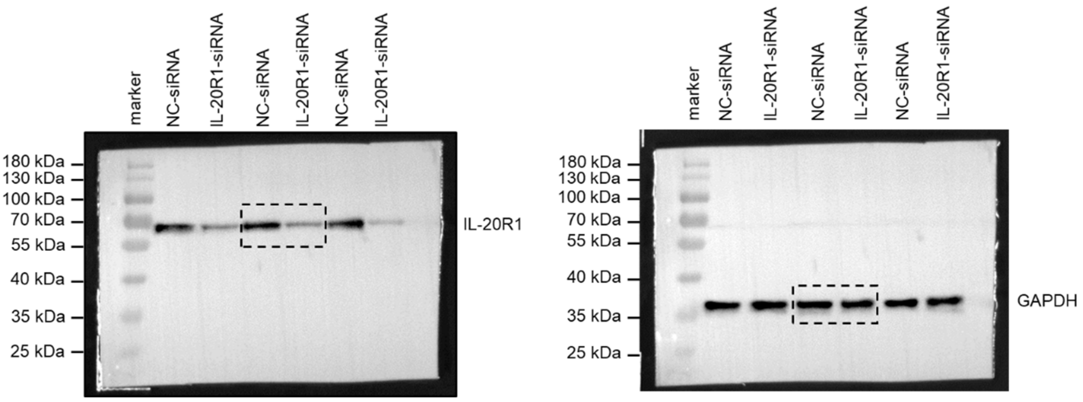

Fig. S3: Intra-TG administration of IL-20R1-siRNA decreased the protein expression of IL-20R1 in TGs. Shown are the full-length images of immunoblots presented in Fig. 2C.

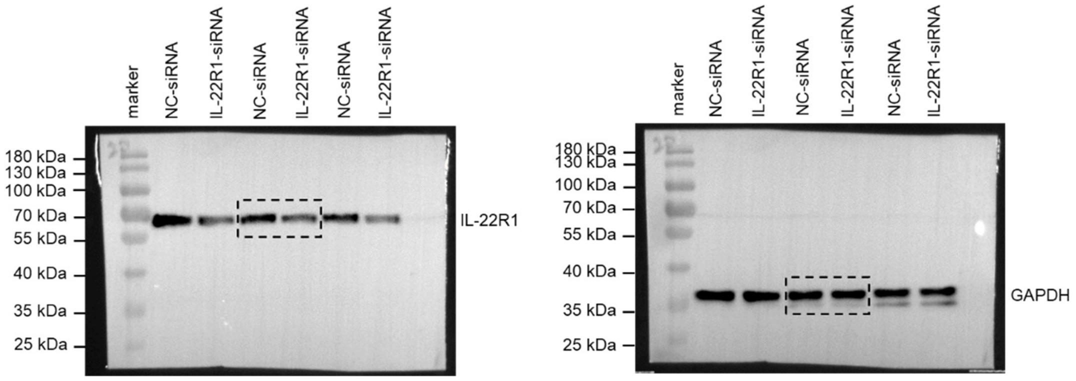

Fig. S4: Intra-TG administration of IL-22R1-siRNA decreased the protein expression of IL-22R1 in TGs. Shown are the full-length images of immunoblots presented in Fig. 2D.

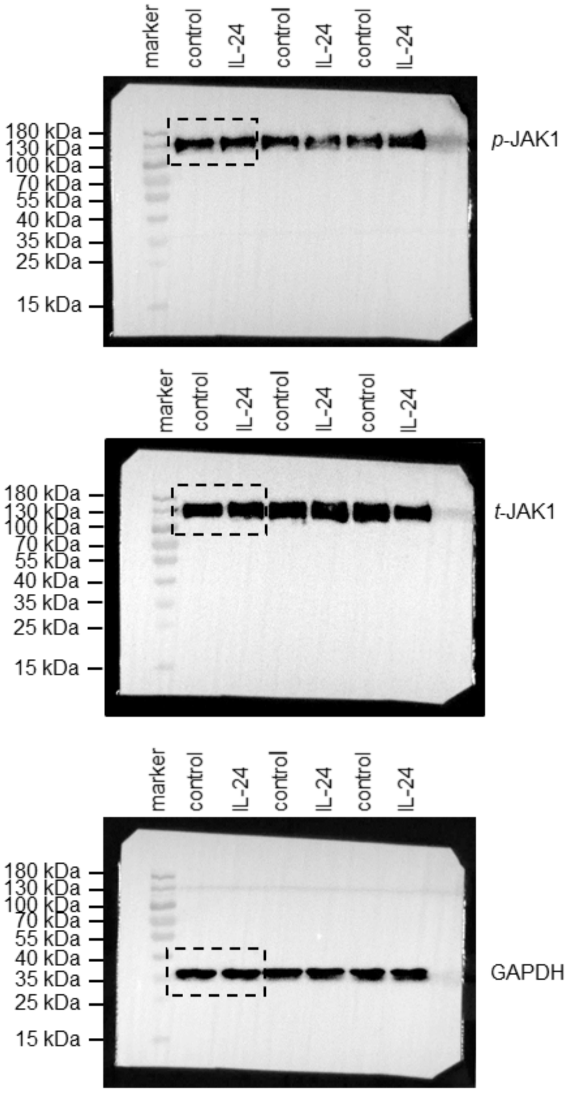

Fig. S5: Protein expression of *p*-JAK1 and *t*-JAK1 in mouse TGs. Shown are the full-length images of immunoblots presented in Fig. 3A.

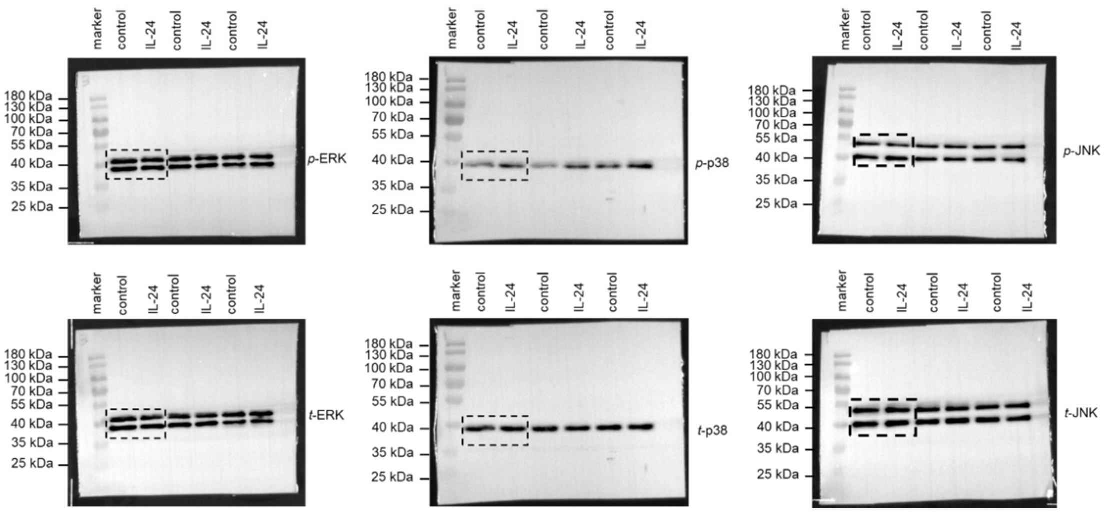

Fig. S6: Protein expression of phosphorylated and total ERK, p38, JNK in mouse TGs. Shown are the full-length images of immunoblots presented in Fig. 3C.

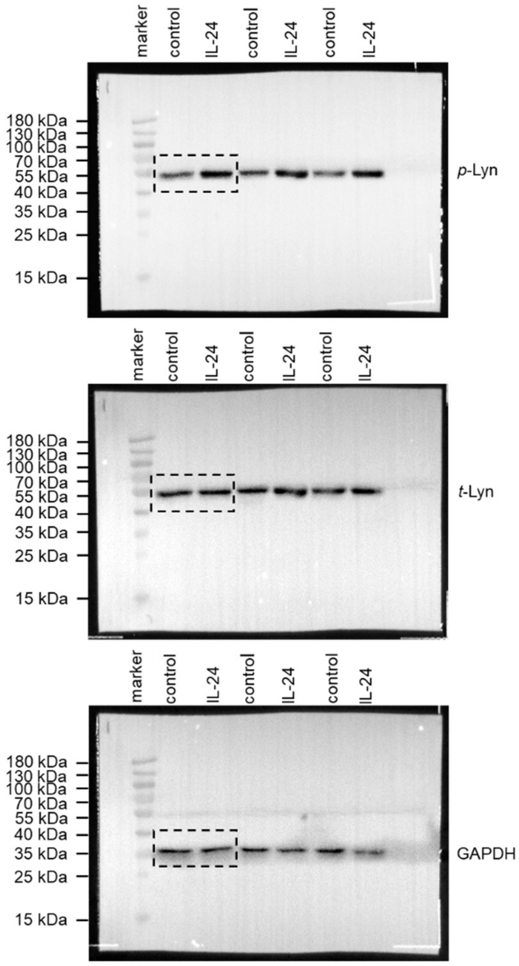

Fig. S7: Protein expression of *p*-Lyn and *t*-Lyn in mouse TGs. Shown are the full-length images of immunoblots presented in Fig. 3E.

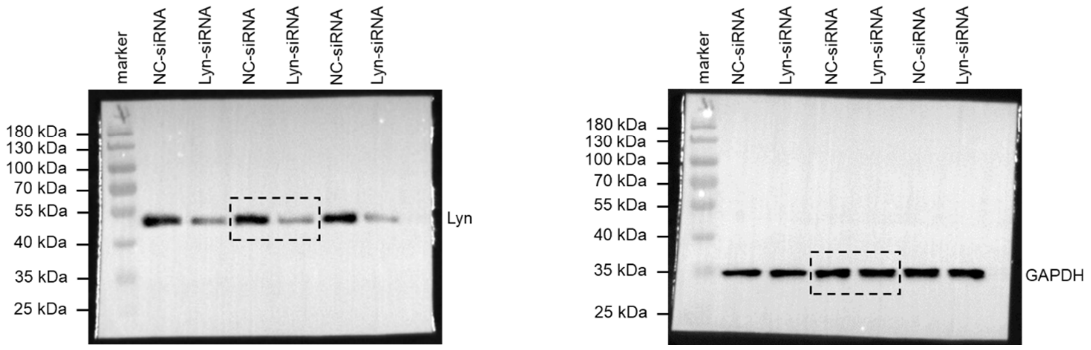

Fig. S8: Intra-TG administration of Lyn-siRNA decreased the protein expression of Lyn in TGs. Shown are the full-length images of immunoblots presented in Fig. 3G.

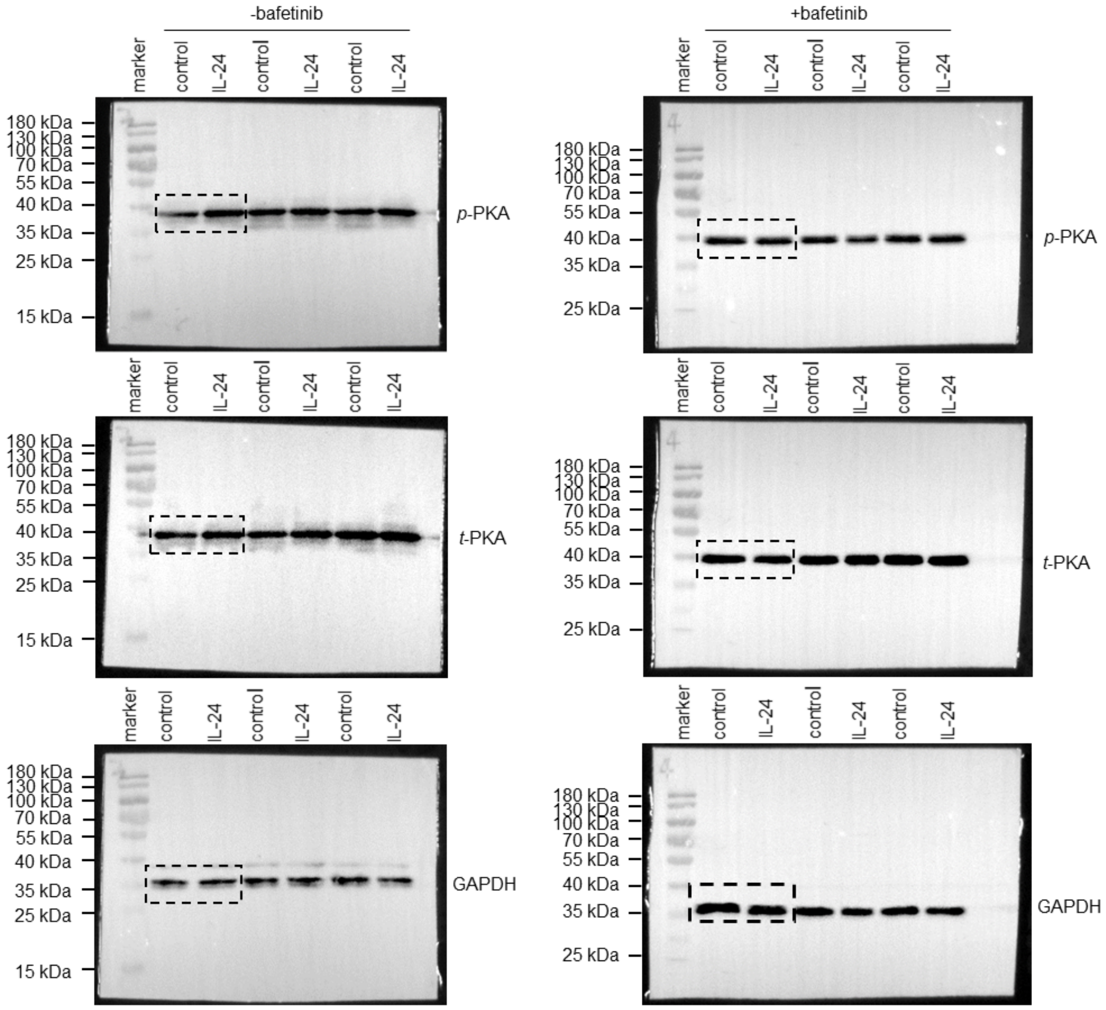

Fig. S9: Analysis of phosphorylated PKA (*p*-PKA, PKA activation) in TG cells upon application of IL-24 (150 ng/ml) with or without pretreatment with bafetinib (1 μM). Shown are the full-length images of immunoblots presented in Fig. 4D.

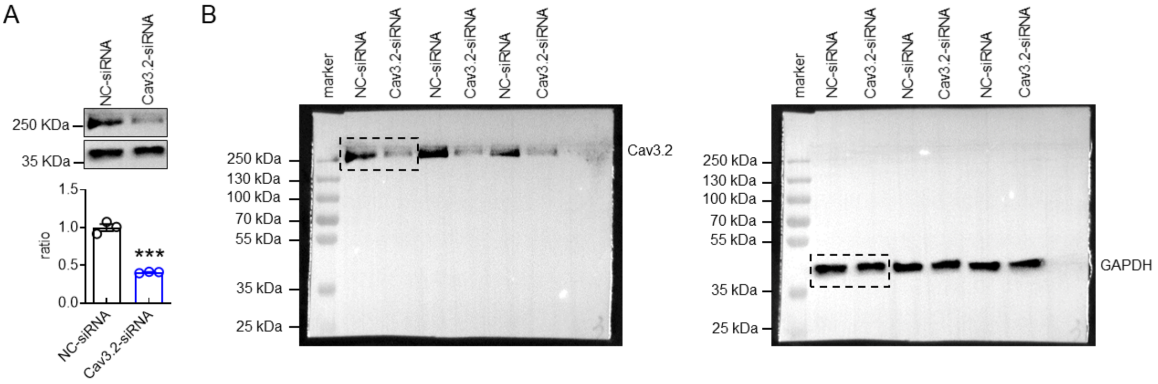

Fig. S10: Knockdown of Cav3.2 in intact mice. **(A)** Protein expression of Cav3.2 in TGs of mice treated with NC-siRNA- or Cav3.2-siRNA. ***p < 0.001 (compared to NC-siRNA, unpaired t-test). **(B)** Shown are the full-length images of immunoblots presented in panel *A*.

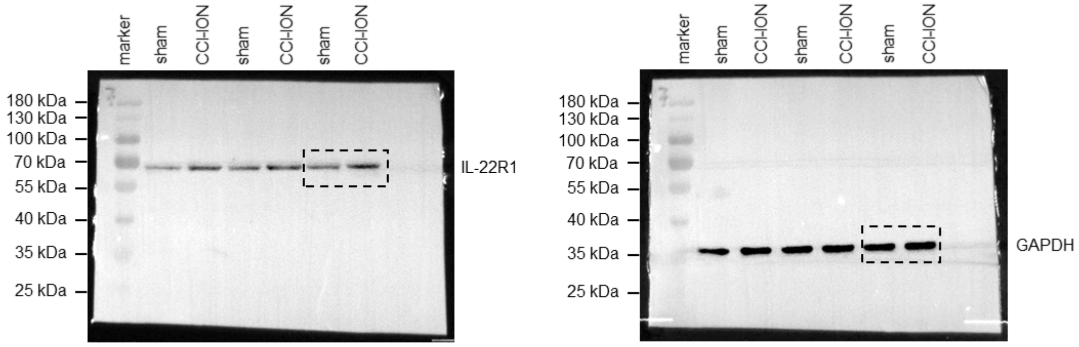

Fig. S11: Immunoblot analysis of IL-22R1 in mouse TGs 14 days after CCI-ION or sham-operation. Shown are the full-length images of immunoblots presented in Fig. 6F.
